# Implicit Metaethical Intuitions: Validating and Employing a New IAT Procedure

**DOI:** 10.1007/s13164-021-00572-3

**Published:** 2021-11-12

**Authors:** Johannes M. J. Wagner, Thomas Pölzler, Jennifer C. Wright

**Affiliations:** 1 Department of Philosophy, University of Graz, Attemsgasse 25/II, 8010 Graz, Austria; 2 Department of Psychology, College of Charleston, Charleston, SC USA

**Keywords:** Metaethical intuitions, Moral objectivism, Implicit attitudes, Implicit meta-ethical intuitions, Metaethical pluralism, Extreme moral issues, ‘Metaethical Intuitions IAT’

## Abstract

Philosophical arguments often assume that the folk tends towards moral objectivism. Although recent psychological studies have indicated that lay persons’ attitudes to morality are best characterized in terms of non-objectivism-leaning pluralism, it has been maintained that the folk may be committed to moral objectivism *implicitly*. Since the studies conducted so far almost exclusively assessed subjects’ metaethical attitudes via explicit cognitions, the strength of this rebuttal remains unclear. The current study attempts to test the folk’s implicit metaethical commitments. We present results of a newly developed Implicit Association Test (IAT) for metaethical attitudes which indicate that the folk generally tend towards moral non-objectivism on the implicit level as well. We discuss implications of this finding for the philosophical debate.

## Introduction

Moral objectivism is a view in metaethics. As such, it does not involve or entail answers to moral questions. Proponents of this view rather hold a metaphysical claim *about* such questions: whatever the correct answers to moral questions, they argue, these answers are *objectively* correct, i.e., they do not depend on what anybody thinks about them. According to moral objectivists, then, there are truths about moral issues, and these truths would hold even if we ourselves or most members of our culture or anybody else thought differently about these issues (e.g., Brink 1989; Huemer 2005; Shafer-Landau 2003, 2006).

So far philosophical debates about moral objectivism have been dominated by arguments against it, such as Mackie’s argument from moral disagreement (Mackie 1977) and Harman’s explanatory redundancy argument (Harman 1977). Objectivists have been primarily concerned with attempting to refute these anti-objectivist arguments (e.g., Huemer 2005; Shafer-Landau 2006). Much more rarely have they developed positive arguments in their favor. This dialectic is explained by the fact that both sides — non-objectivists as well as objectivists — accept that we have a prima facie reason to believe in objective moral truths. In other words, metaethics proceeds under the assumption that non-objectivists bear the “burden of proof”; that as long as they haven’t presented strong arguments in favor of their view, we cannot be justified in adopting it (e.g., Blackburn 2006; Brink 1989; Dancy 1986; Huemer 2005; Mackie 1977).

Why is this? The case for non-objectivists’ burden of proof typically starts from an empirical assumption: the observation that objectivism reflects lay people’s intuitions about morality. This observation appears to be best explained or justified on objectivist grounds. For example, it has been claimed that the best explanation of why lay people have the intuition that there are objective moral truths simply is that there are such truths. Hence, the argument goes, we have a prima facie reason to accept objectivism (e.g., Brink 1989; Dancy 1986; Huemer 2005).

This paper concerns the underlying empirical assumption of the above “burden of proof argument”. In recent years psychological research has challenged that lay people tend towards objectivism. While early investigations into folk metaethics seem to have supported this observation (e.g., Beebe and Sackris 2016; Beebe et al. 2015; Goodwin and Darley 2008, 2012; Nichols 2004; Nichols and Folds-Bennett 2003; Wainryb et al. 2004), once certain methodological improvements had been implemented, research rather started to converge on the hypothesis of folk metaethical pluralism; in particular, non-objectivism-leaning pluralism. That is, lay people have been found to hold that while some moral questions do admit of objectively correct answers, most of them do not (e.g., Davis 2021; Pölzler and Wright 2020; Sarkissian et al. 2011; Theriault et al. 2017; Wright and Pölzler forthcoming; see also Hopster 2019; Pölzler 2017; Wright et al. 2013; Wright et al. 2014).

The main way in which moral objectivists have responded to criticism of the burden of proof argument’s empirical assumption is to argue that this criticism focuses on the wrong kind of intuitions. Studies such as those mentioned above seem to be about how people *explicitly* think about metaethics. However, the intuitions that are relevant to philosophy — and to the burden of proof argument for moral objectivism in particular — have rather been claimed to be *implicit*, in the sense that they underlie and manifest themselves in moral thought, discourse and behavior without people generally being consciously aware of them. After all, the argument goes, given the abstractness and complexity of metaethics, people are likely to misinterpret or err about their own intuitions (e.g., Björnsson 2012; Brink 1989; Enoch 2005, 2014; for discussion see Zijlstra forthcoming).^1^ Enoch, for example, writes:

[W]hat is relevant is not the explicit metanormative beliefs – much less the explicit metanormative statements – of participants in normative discourse. What is relevant, rather, are the deep metanormative commitments embedded (perhaps implicitly) in normative discourse and practice themselves. The fact that many sophomores (and not only them) express some subjectivist or relativist metanormative intuitions thus has very little weight in assessing the commitments of normative discourse. (Enoch 2005: 773, footnote 31)

If objectivists are right that only implicit metaethical intuitions matter, then their burden of proof argument is indeed largely immune against recent empirical challenges; for only very few studies on folk metaethics might be plausibly interpreted as having investigated intuitions of this kind (e.g., Pölzler et al. under review; Theriault et al. 2017; Zijlstra forthcoming). Moreover, even these studies might have triggered only *somewhat* implicit metaethical thinking or might be subject to other methodological worries.^2^ Our study aims to contribute to filling this research gap. As will be reported in the present paper, we piloted a novel measure of implicit intuitions about the objectivity of morality, thus aiming to empirically test moral objectivists’ assumption of implicit folk objectivism.

Our novel measure of implicit metaethical intuitions is a form of *Implicit Association Test*, or IAT (Greenwald et al. 1998), that we will refer to as the ‘Metaethical Intuitions IAT’. We are aware of recent psychometric criticisms of tests of this kind (Forscher et al. 2019; Fiedler et al. 2006). Yet, not only have convincing counterarguments to the general criticisms been advanced (Greenwald et al. 2015), but some of them, such as low predictive power of implicit attitudes for behaviors, simply are not relevant in the specific context of broadening our understanding of folk metaethical attitudes.^3^ Thus, while we acknowledge that there is a general debate surrounding the IAT rationale, we rely on the fact that IATs remain a well-established, widely accepted and much researched procedure in social psychology. With their focus on unconscious associations, the IAT procedure seems a particularly well-suited tool for measuring the “deep” metaethical intuitions that philosophers such as Enoch have speculated about.

In developing an IAT assessment of metaethical intuitions we followed a domain-based approach. Specifically, we based our task design on the idea that objectivist or subjectivist metaethical intuitions are indicated depending on whether moral concepts are associated with terms from other ‘objective’ or ‘subjective’ domains. The ‘Metaethical Intuitions IAT’ procedure we propose relies on using the following paradigmatic domain associations to infer metaethical intuitions: If moral concepts are rather associated with the domain of science,^4^ we take this to indicate the metaethical attitude of moral objectivism; if moral concepts are perceived as being closer to the domains of personal preference or social conventions, we ascribe the metaethical attitude of moral subjectivism.

In addition to our newly developed implicit measure, we also assessed explicit metaethical attitudes with tasks routinely used in research on folk metaethical intuitions. Using more established measures enabled us to conduct a comprehensive comparison of implicit and explicit result patterns. However, our study did not aim to establish causal or unidirectionally predictive links between layers of attitudes at this early stage of development. We were mainly interested in testing associations and parallel patterns between implicit and explicit attitudes, as we assume them to be at least weakly related subcomponents of the more general construct of folk metaethical intuitions. Under this assumption, we expect and interpret corresponding patterns and associations as prima facie evidence for convergent validity (Strauss and Smith 2009; Campbell and Fiske 1959). By comparing and contrasting our IAT results with various aspects of these more established explicit procedures, we intend to prepare the grounds for further development and full validation in future studies.

In what follows we first state the hypotheses that we set out to test. We then explain the set-up of the newly developed ‘Metaethical Intuitions IAT’ as well as the explicit measures of metaethical intuitions we employed. Finally, we present our study’s results and discuss their implications both for the validation of the ‘Metaethical Intuitions IAT’ and the question of lay people’s implicit metaethical intuitions. First evidence from a 213-participant sample supports the validity of the ‘Metaethical Intuitions IAT’ and that also on an implicit level, lay people’s intuitions dominantly manifest as non-objectivism-leaning. We conclude that this undermines objectivists’ response to the empirical challenge against their burden of proof argument.

### Hypotheses

We formulate the following four sets of hypotheses, which we preregistered in advance of collecting any data (Pölzler et al. 2019):

### A. Implicit Level

As has been mentioned, much recent psychological research found non-objectivism to be dominant on the explicit level (e.g., Sarkissian et al. 2011; Pölzler and Wright 2020; Wright and Pölzler forthcoming). We therefore expect to find it to be dominant with implicit metaethical intuitions as well. This should manifest in IAT results as the domain of *Morality*^5^ being more closely associated with prototypical ‘subjective’ domains than with the paradigmatically ‘objective’ domain of *Science*. This expectation is also consistent with Theriault et al. (2017), who found that people neurologically process morality more like a set of preferences than a set of facts.

- (A1) Thus, we hypothesize that people implicitly lean towards moral non-objectivism. Specifically, we expect a stronger implicit association of *Morality* and *Personal Preference* rather than of *Morality* and *Science*, as well as a stronger implicit association of *Morality* and *Social Convention* rather than of *Morality* and *Science*.
- (A2) Moreover, considering research on the explicit level (e.g., Goodwin and Darley 2008; Wright et al. 2013; Wright and Pölzler forthcoming), we also hypothesize that more ‘extreme’ moral issues (such as rape or genocide) trigger a higher ratio of implicit objectivist intuitions. Specifically, we expect that these extreme items will show stronger implicit associations with *Science*, and thus indicate objectivist moral intuitions.

### B. Explicit Level

Our hypotheses regarding explicit metaethical intuitions draw on previous studies in this area.

- (B1) Consistent with past findings (e.g., Sarkissian et al. 2011; Pölzler and Wright 2020; Wright and Pölzler forthcoming), we expect overall non-objectivism on the explicit level regarding the domain of *Morality*.
- (B2) However, we hypothesize that the ‘extreme’ moral items mentioned above will actually induce a higher ratio of objectivist responses on the explicit level (e.g., Goodwin and Darley 2008; Wright et al. 2013; Wright and Pölzler forthcoming).
- (B3) In contrast to some previous research in folk metaethics (and in line with Wright et al. 2013), we also assess whether the moral items used in some of our test modalities are classified as moral by subjects themselves as well. Given the contested nature of the boundaries of the domain of *Morality* (Wright et al. 2013), our hypothesis in this regard is that the ratio of explicit objectivist to non-objectivist responses will change significantly compared to when our a priori classification of scenarios is used (i.e. we pose a non-directional hypothesis).

### C. Relation between Implicit and Explicit Levels

We expect a close associative relation between implicit and explicit metaethical intuitions.

- (C1) Specifically, we hypothesize that the more participants lean towards moral objectivism (i.e. associating *Morality* with *Science*) on the explicit level, the more they also lean towards moral objectivism on the implicit level.
- (C2) We expect a parallel pattern of results on implicit and explicit levels. This should manifest as a general non-objectivist leaning pluralism on the explicit and implicit level, with ‘extreme’ moral items eliciting a higher ratio of objectivist attitudes, also on both levels.^6^

### D. Consistency of Metaethical Intuitions

Prior research on folk metaethics suggests that on the explicit level different moral scenarios elicit different metaethical intuitions (e.g., Goodwin and Darley 2008, 2012; Davis 2021; Wright et al. 2013; Wright and Pölzler forthcoming; Wright 2018). We therefore expect

- (D1) low intra-individual consistency of metaethical intuitions, as well as
- (D2) low inter-item consistency with respect to the domain of *Morality*.

## Method

### Participants

#### Launching Platform and Reimbursement

We recruited participants through Amazon Mechanical Turk (M-Turk), advertising a reimbursement of 3.50$ for roughly 30 min.^7^

#### Exclusion of Data

We received complete data from 260 participants. Within this sample, we excluded participants who (a) did not sufficiently complete the ‘explicit’ tasks or rushed through the entire study in less than 15 min; or (b) missed more than two of the four attention checks inserted among the ‘explicit’ task questions – simple statements which can be easily answered if attentively read, such as “please select the Nr. 4 below”, (see Pölzler forthcoming); or (c) had an error rate of 20% percent or higher in the ‘implicit’ tasks (a criterion adopted from Karpinski and Steinman 2006).^8^ These exclusion criteria were preregistered prior to data collection (Pölzler et al. 2019). On their basis we excluded 47 participants.

#### Sample

We conducted our analyses on data of a sample of 213 participants. Overall, the participants were 55% male; 77% White/Caucasian, 10% Black/African-American, 7% Asian/Pacific Islander, and 3% Hispanic/Latino, with an age range of 20–72 years, *M* = 39.9, *SD* = 12.0.

### Implicit Tasks: The ‘Metaethical Intuitions IAT’

#### Millisecond

We administered the IAT segment of our study on the platform “Millisecond”, using a web-license on www.millisecond.com that operates Millisecond’s experimental-paradigm program ‘Inquisit (5.0.14.0)’.^9^

#### Task Design: Single Category Implicit Association Test for Implicit Metaethical Intuitions

We attempted to measure implicit metaethical intuitions using a standard testing paradigm for implicit attitudes, the *Implicit Association Test* (IAT; Greenwald et al. 1998). The IAT is a widely used cognitive-behavioral paradigm that purports to measure the strength of automatic (implicit) associations between concepts in people’s minds on the basis of latencies in a simple sorting task. In line with IAT conventions, we calculated *d*-scores using the improved scoring algorithm as described in Greenwald et al. (2003).

We adapted the IAT procedure given our research interest, i.e., the question of whether people understand moral concepts as objective or subjective. Taken by themselves, the concept pairing ‘objective/subjective’ might be too technical and ambiguous for a sample untrained in the terminology of academic philosophy. We therefore decided to measure participants’ implicit metaethical intuitions by investigating how they associate moral concepts with concepts from other *domains* that we took to be paradigmatically subjective or objective. The two domains that we took to be clearly subjective in nature were *Personal Preferences* and *Social Conventions*. The domain which we took to be paradigmatically objective was *Science*. We think that observing whether the domain *Morality* falls more closely to the *Personal Preference* / *Social Convention* or to the *Science* end of the spectrum should allow us to make inferences about *Morality’s* implicit association with subjectivity or objectivity; that is, this type of observation should be indicative of implicit metaethical cognition.

Due to this set-up, the core concepts underpinning our research are taken to represent *domains*, an approach that we adopt from social domain theory, particularly Nucci’s (1981) study (but also Nucci and Turiel 1978), in which the personal, social-conventional and moral were treated as distinct domain categories. More recently, Theriault et al. (2017) employed a domain-based approach to examine metaethical intuitions specifically.

We take a domain to be a field of semantically related terms and propositional contents that collectively tend to land somewhere on the spectrum of subjectivity/objectivity as people consider their epistemic status. Each of the four domains that we employed for the IAT task dimensions was operationalized through 12 typical items. We operationalized the domain of *Morality* by using concepts that capture the diverse aspects of the domain, such as duties, virtues and vices, good or bad actions, values, harm, extreme deliberate injuries etc.^10^

In particular, the items of the moral domain were ‘doing good deeds’, ‘injustice’, ‘inflicting pain’, ‘helping the needy’, ‘ethical values’, ‘vicious’, ‘being honest’, ‘bravery’, ‘expressing empathy’, ‘rape’, ‘genocide’ and ‘acting generously’. Examples for concepts in the other domains are ‘conducting measurements’ or ‘reliable methods’ for *Science*; ‘favorite food’ or ‘taste in music’ for *Personal Preference*; ‘being polite’ or ‘table manners’ for *Social Convention* (see appendix for the full list of items by domain).

Moral non-objectivism can take many different forms (Joyce 2021; Pölzler 2018). This is why we decided to test *Morality’s* implicit association not only with one but with two ‘subjective’ domains, *Personal Preference* and *Social Convention*. Hence, we developed one IAT task testing the association of the target domain of *Morality* (M) with the contrast domains of *Social Convention* (SC) vs. *Science* (S) – the ‘SC-M-S IAT’; and a second IAT task testing the association of the target domain of *Morality* (M) with the contrast domains of *Personal Preference* (PP) vs. *Science* (S) – the ‘PP-M-S IAT’.

People might associate *Morality* with one of the above-mentioned domains for different reasons. To prompt implicit cognitions about the particular feature that interested us (subjectivity/objectivity), we presented the label “Subjective” in brackets whenever we used the concepts “Social Convention” and “Personal Preference”, and appended the label “Objective” in brackets whenever we displayed the concept “Science”. Our two IAT tasks — both so-called ‘Single-Category IATs’, since their IAT target dimension contains only a single concept (Karpinski and Steinman 2006)^11^ — were thus comprised of the dimensions and labels displayed in Table 1. For further illustration, Table 2 provides a display of the task rationale of one of our IAT tasks, the SC-M-S IAT (the structure of the PP-M-S IAT task was analogous).

**Table 1:** IAT tasks: dimensions and labels

**IAT 1: Social Convention – Morality – Science (SC-M-S IAT)**		
*Target dimension:*	(1) Morality	
*Attributive dimension:*	(1) Social Convention (Subjective)	(2) Science (Objective)
**IAT 2: Personal Preference – Morality – Science (PP-M-S IAT)**		
*Target dimension:*	(1) Morality	
*Attributive dimension:*	(1) Personal Preference (Subjective)	(2) Science (Objective)

**Table 2:**
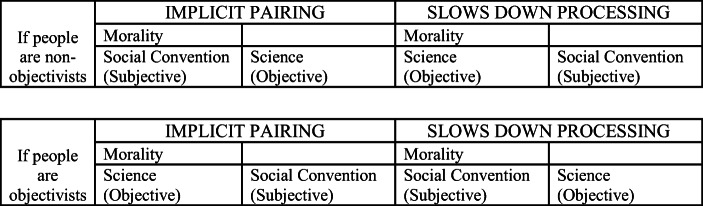
Task rationale, using the example of the SC-M-S IAT

**Fig. 1:**
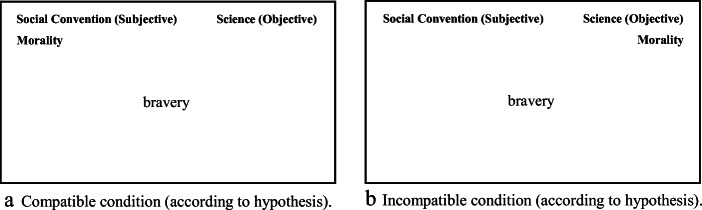
Placement of domains for compatible and incompatible conditions – using the SC-M-S IAT as example

*IAT task set-up.* The two IAT tasks had an identical structure, so it will suffice to explain the set-up with reference to the SC-M-S IAT. In each condition of the IAT, participants were asked to categorize items into three domains indicated by labels on top of the screen: “Morality”, “Social Convention (Subjective)” (which, in the PP-M-S IAT, was replaced by “Personal Preference (Subjective)”), “Science (Objective)”. As in any IAT procedure, there was one compatible and one incompatible condition, a distinction based on researchers’ expectations regarding the direction of the resulting IAT effect. Since we hypothesized effects leaning towards non-objectivism, the compatible set-up was defined as the constellation that grouped *Morality* and one of the two ‘subjective’ domains on one side. Thus, in the compatible set-up of labels displayed in Fig. 1a, participants were supposed to press the left key (E) if the item belonged to one of the domains whose labels were presented on the left (e.g. “Morality” OR “Social Convention (Subjective)”) or to press the right key (I) if the item belonged to the domain whose label was presented on the right (e.g. “Science (Objective)”). Domain label pairings are reversed in the incompatible label configuration (see Fig. 1b), such that participants are supposed to press the left key (E) if an item belonged to the domain presented on the left (e.g. “Social Convention (Subjective)”) and to press the right key (I) if the item belonged to one of the domains presented on the right (“Science (Objective)” OR “Morality”). Before each of the two trials, a practice trial had to be completed in order for participants to get used to the domain label configuration. Participants were required to self-correct responses through error feedback to remove any ambiguity regarding which item belongs to what domain. No error feedback was provided in experimental blocks.

#### Counterbalancing of IAT Factors

In general, the IAT segment of the study was administered prior to the explicit tasks in order to avoid prompting explicit metaethical reflections during the IAT tasks. Within the IAT segment, three factors were counterbalanced: (1) The order of presenting either the SC-M-S or the PP-M-S IAT task first was counterbalanced across participants. (2) Whether the label “Morality” appeared first on the left or first on the right side of the screen was counterbalanced, for each IAT, across participants.^12^ (3) Whether the hypothesis-consistent or hypothesis-inconsistent label configuration would be presented first or second was counterbalanced across participants.

#### Blocks, trials and scoring of IATs

Every participant completed both IAT tasks, each of which had two conditions (compatible and incompatible). The experimental block of each condition was preceded by a block-specific instruction and a practice block of 36 trials (drawing all 12 items from each of the three IAT-specific domains, with items drawn randomly from the respective domain pools with no repetition). The experimental block in each condition consisted of 84 trials. Although items were presented in randomized order, all participants would work through the same set of 84 items pertaining to that IAT condition. Following Karpinski and Steinman (2006), the 1:2 category label set-up of a single-category IAT requires uneven numbers of item presentation across domains in order to accommodate left-right imbalance. We adopted a 2:2:3 domain ratio so that 57% of correct responses were on one key and 43% of correct responses were on the other key. Thus, the set of 84 items of one condition consisted of (a) 36 items pertaining to the attribute domain whose category label was presented singularly on one side of the screen (each item of this domain pool appearing exactly 3 times); and (b) 48 items from the other attribute domain and the target domain “Morality” (i.e. 24 items each) whose category labels were presented jointly on the other side of the screen (each item from those domain pools appearing exactly 2 times). The interstimulus-interval was set to 250 ms. In the calculation of the *d*-score for a ‘Single Category IAT’, we followed the procedures outlined by Karpinski and Steinman (2006) as well as Greenwald et al. (2003).^13^

### Explicit Tasks

In order to allow a comprehensive assessment of explicit metaethical intuitions (Pölzler and Wright 2020), we included four tasks in the section of our study that tested attitudes through explicit reflections on scenarios or the meaning of words (= the ‘explicit’ tasks): (1) a *Metaphor* task, (2) a *Disagreement* task, (3) a *Theory 1* task, and (4) a *Theory 2* task. Each of these tasks involved three response types, one of which corresponded to objectivism about the domain in question, one to subjectivist versions of non-objectivism, and one to non-cognitivist versions of non-objectivism (for an explanation of these positions see Pölzler 2018).

#### Administration and Counterbalancing of Explicit Tasks

The explicit tasks were administered online through the survey platform ‘Qualtrics’. Each participant had to complete all of the tasks, i.e., *Metaphor*, *Disagreement*, *Theory 1* and *Theory 2*, in this sequence. The order of scenarios within each task was randomized.

At the end of the survey, we asked participants themselves to sort and classify all scenario questions into the four domains of *Morality*, *Science*, *Social Convention* and *Personal Preference*. We then used each participant’s classification of scenarios in a further analysis to re-score their metaethical intuitions for each domain, in order to assess potential significant contrasts between self- and researcher-based scenario classifications.

#### Questions Used to Construct Scenarios

In the *Metaphor*, *Disagreement*, and *Theory 1* task modalities, we used the same nine basic questions to construct their scenarios. Each of these questions we considered to be typical for a particular domain: We employed three *Morality* questions, two *Personal Preference* questions, two *Social Convention* questions, and two *Science* questions. Parallel to the items on the implicit level, the three morality questions included an ‘extreme’ moral questions, namely ‘Is raping a woman wrong?’. The two ‘non-extreme’ moral questions were ‘Was Martin Luther King admiringly brave?’ and ‘Do we have a moral duty to help the needy?’. The non-moral questions were as follows: *Science*, ‘Is the chemical formula of water H20?’, ‘Are brain scans a reliable scientific method?’; *Personal Preference*, ‘Was Mozart a better musician than is Lady Gaga?’, ‘Do socks and sandals look terrible?’; *Social Convention*, ‘Ought one to speak with one’s mouth full?’, ‘Is it wrong to wear pajamas to a seminar meeting?’ In every task modality, each of these questions was incorporated into a scenario which was constructed according to the specifics of that task modality. We will now briefly describe the rationale of each of the four explicit task modalities.

#### (1) Metaphor Task

Moral objectivism and non-objectivism are claims about moral facts. Our metaphor task (adapted from Pölzler and Wright 2020) asked participants to choose between a number of metaphors for these facts which correspond to objectivism and the above-mentioned variants of non-objectivism. For example, with regard to ‘Do we have a moral duty to help the needy’ the task read as follows:

Do we have a moral duty to help the needy? Here we are not interested in what is the correct answer to this question. Rather, we will present you with metaphors about whether there is a correct answer to the question, and if yes, what it is that makes this answer correct.

Which of these metaphors seems most appropriate to you?

- There is a correct answer to this question. It is “out there in the world”. (1)
- There is a correct answer to this question. It is “invented or created by individuals or societies”. (2)
- There is not a correct answer to this question. Any response to the question is just like someone saying “Booh!” or “Hooray!” (3)

Responses of the first kind were interpreted as being indicative of explicit intuitions in favor of objectivity about the target question, while answers of the second and third kind were taken as indicative of non-objectivism.

#### (2) Disagreement Task

Disagreement tasks have been the most popular way of trying to measure folk moral objectivism and have been used, e.g., by Beebe et al. 2015; Beebe and Sackris 2016; Goodwin and Darley 2008, 2012; Nichols 2004; Sarkissian et al. 2011 and Wainryb et al. 2004. In our version of the disagreement task (adapted from Wright and Pölzler forthcoming), participants were presented with two cases of disagreement (A + B) and were asked to interpret these cases. Case A presented intracultural disagreement.

Consider the following situation. Two people who are members of the same culture or community discuss whether we have a moral duty to help the needy. One person says that we have a moral duty to help the needy. The other person says that we do not have a moral duty to help the needy.

Which interpretation of this disagreement seems most appropriate to you?

- One of these two people is correct and the other one is incorrect (1)
- Both people are correct (2)
- Neither person is correct nor incorrect (3)

Case B was identical, only that this time the disagreeing parties were said to be members of *different* cultures or communities, with each of their judgements conforming to the dominant view within their culture or community. Participants who chose the first answer option both with regard to A and B were scored as explicit objectivists about the question at issue. Other response combinations were either scored as non-objectivist or as non-standard.

#### (3) Theory 1

Our first theory task (adapted from Pölzler and Wright 2020) asked subjects to choose among theoretical descriptions that correspond to objectivism and versions of non-objectivism.

Do we have a moral duty to help the needy? Here we are not interested in what is the correct answer to this question. Rather, we will present you with theories about whether there is such an answer, and if yes, what makes this answer correct.

Which of these theories seems most appropriate to you?

- There is a correct answer to the above question. It successfully represents a fact. This fact is objective, i.e., independent from what anybody thinks about it. In other words, even if an individual or society were to regard the answer as incorrect it would still be correct. (1)
- There is a correct answer to the above question. It successfully represents a fact. This fact is subjective, i.e., depends on what individuals or societies think about it. In other words, if an individual or society were to regard the answer as incorrect then this would make the answer incorrect (for them). (2)
- There is not a correct answer to the above question at all. Rather than (successfully) representing a fact, any answer to this question just expresses a person’s feelings, intentions, emotions or attitudes about it. (3)

As with the metaphor task, the first response was interpreted as indicating explicit objectivist intuitions, while the second and third response was interpreted as being indicative of non-objectivism.

#### (4) Theory 2

Finally, we developed a new theory task that enabled us to measure participants’ explicit metaethical intuitions about the very same items that we used in the implicit part of the study, thus increasing comparability between the two levels. With regard to the domain of *Morality*, for instance, we presented the list of ‘moral’ items (as used in the IAT task and displayed in the appendix) and had participants rate the following statements on a 9-point Likert-scale (from ‘strongly agree’ to ‘strongly disagree’):

- I think that these terms, by and large, refer to something objective, real, and rooted in the world or describe ways of how we find out objective truths. The terms can hence be used to state objective truths about particular situations. They are vital in understanding reality. (A)
- I think that these terms, by and large, refer to something subjective that is rooted in ourselves or our communities or describe ways of how we find out subjective truths. The terms can hence be used to state subjective truths about particular situations. They are vital in understanding ourselves or our communities. (B)
- I think that these terms, by and large, neither refer to something objective nor to something subjective. They are rather ways of expressing the feelings, intentions, emotions or attitudes that we have towards certain things, of persuading others and getting them to behave in certain ways. The terms are vital for these practical purposes. (C)

We generally took the response to (A) as indicative of objectivism, and (B) and (C) as non-objectivist responses with respect to the domain in question. We attempted, however, to employ a metric that would interpret the responses on the objectivist scale relative to the average of the non-objectivist responses. To this end, we converted the Likert-scale response into numeric values from 0 to 8 and calculated percentage scores for domain objectivism according to the following formula:

$$ \frac{(A)-\frac{(B)+(C)}{2}+8}{16} $$

**Fig. 2:**
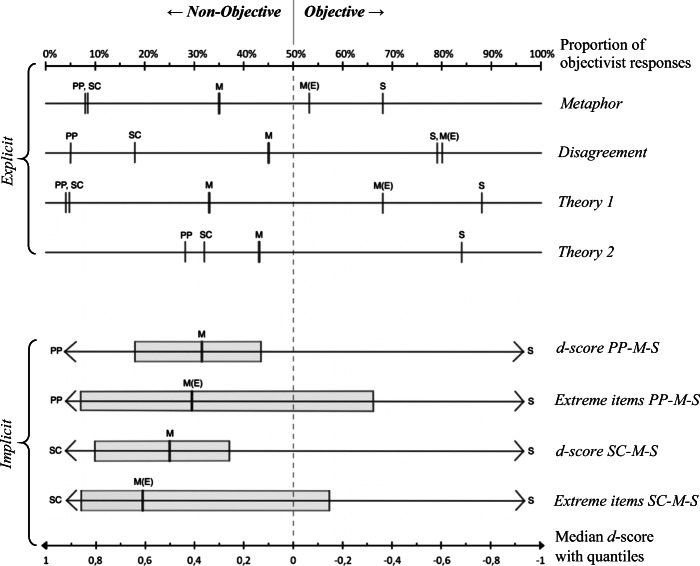
Personal Preference – Social Convention – Morality – Science: Objectivist/non-objectivist intuitions in four domains on explicit and implicit levels. *Note:* Explicit and implicit measures are plotted with their respective x-axis. For the explicit level, the percentage of objectivist answers in each domain has been plotted for each of the explicit tasks. Based on the arithmetic mean of objectivist responses, every domain (M = *Morality*, S = *Science*, PP = *Personal Preference*, SC = *Social Convention*) has been located on the objective/non-objective spectrum of each task modality. Also, for those three task modalities in which an extreme scenario was used, a value for the metaethical intuitions regarding only the extreme scenario was calculated, M(E) = *‘Extreme’ moral items*. In the lower part of the figure, the implicit measures (IAT *d*-scores) are plotted analogously, such that the x-axis of *d*-scores is matched with the above x-axis of explicit task modalities on the differing objective/non-objective cut-off value. However, while it was possible to locate the domains *Personal Preference / Social Convention* and *Science* on the objective/non-objective spectrum for the explicit tasks, this is not wholly possible with respect to the implicit tasks, as *d*-scores resultant of the IATs make only indirect reference to these domains by indicating whether the domain of *Morality* is tendentially rather associated with one pole or the other. Because the *d*-score is in principle open in both directions of the scale, the two reference domains of each IAT (PP-S or SC-S) have been placed as ‘limit’ at their respective end of the *d*-score spectrum. *d*-score distributions are represented by the median with lower and upper quantiles. Also, local *d*-scores of extreme items are plotted separately for each IAT. Regarding explicit measures: All differences between domains are significant at *p* < .05, unless indicated by domain letters which are grouped together and merely separated by a comma. ‘Extreme’ moral items (indicator “M(E)”) have not been included in statistical comparisons between domains but have been tested individually against the cut-off value of 50%.

The resulting distribution ranged from 0 to 1, with .5 as the cut-off value between non-objectivism and objectivism. We used this score for percentage comparisons with other explicit tasks (see, for instance, Fig. 2).

## Results

In this section we will present our study’s results, beginning with the implicit tasks (Hypotheses A), moving on to the explicit tasks (Hypotheses B) and the relation between the implicit and explicit tasks (Hypotheses C), finally presenting findings regarding the consistency of metaethical intuitions and their intra-individual and inter-scenario stability (Hypotheses D).

### Implicit Level (A) – The ‘Metaethical Intuitions IAT’

#### (A1) Moral Objectivism vs. Non-objectivism

Split-half reliability (Spearman-Brown corrected) was good for both IAT tasks, *r* = .85 for the SC-M-S IAT and *r* = .85 for the PP-M-S IAT. All *d*-scores reported in this section are graphically displayed in Fig. 2. The PP-M-S IAT yielded a *d*-score of .37 (*SD* = 0.35), and a one sample *t*-test established that this *d*-score was different from 0, *t*(212) = 15.40, *p* < .01, thus demonstrating that the domain of *Morality* was more strongly associated with the ‘subjective’ domain of *Personal Preference* rather than the ‘objective’ domain of *Science*. The SC-M-S IAT yielded a *d*-score of .52 (*SD* = 0.37) and a one sample *t*-test established that this *d*-score was different from 0, *t*(212) = 20.42, *p* < .01, thus demonstrating that the domain of *Morality* was more strongly associated with the ‘subjective’ domain of *Social Convention* rather than the ‘objective’ domain of *Science*. Comparing the general *d*-scores of the two IATs showed that the association of *Morality* with *Social Convention* was relatively stronger than the association of *Morality* and *Personal Preference*, *t*(212) = 4.98, *p* < .01. Generalizing across both single-category IATs, we may say that participants displayed a stronger association of the domain of *Morality* with the subjective domains of *Personal Preference* and *Social Convention* than with the objective domain of *Science*. On the assumption that these tasks have sufficient validity, this is an indication that even in their implicit cognitions people tend towards moral non-objectivism.^14^

#### (A2) 'Extreme' Moral Items

Concordant with this general pattern but contrary to our hypothesis (A2), local *d*-score calculations showed that the extreme items ‘genocide’ and ‘rape’ also exhibited non-objectivist metaethical intuitions on the implicit level (see also Fig. 2).^15^ The PP-M-S IAT yielded a local *d*-score for ‘extreme’ items of .41 (*SD* = 0.79), and a one sample *t*-test established that this *d*-score was different from 0, *t*(209) = 7.44, *p* < .01, indicating that extreme moral items were more strongly associated with the ‘subjective’ domain of *Personal Preference* than with the ‘objective’ domain of *Science*. An analogous finding emerged for the SC-M-S IAT, which yielded a local *d*-score for ‘extreme’ items of .62 (*SD* = 0.76). A one sample *t*-test established this *d*-score to be different from 0, *t*(208) = 11.83, *p* < .01, thus indicating that extreme moral items were more strongly associated with the ‘subjective’ domain of *Social Convention* rather than the ‘objective’ domain of *Science*. These non-objectivist results for the ‘extreme’ IAT items were contrary to our hypothesis A2, which we had formulated with reference to past studies which had found that more extreme issues elicit more objectivist responses in explicit tasks.

### Explicit Level (B)

#### (B1) Moral Objectivism vs. Non-objectivism

Figure 2 displays the average ratio of objectivist/non-objectivist intuitions for each domain in each task modality, the results that will now be discussed in detail. The objectivist/non-objectivist ratio ranges from 0% (=none of the intuitions was objectivist) to 100% (all intuitions were objectivist). Since for these analyses all intuitions were categorized as either objectivist or non-objectivist, the cut-off value of 50% signifies an exact balance in the ratio of objectivist to non-objectivist intuitions.

To determine whether there were significant differences between objectivist/non-objectivist ratios among domains, we ran a one-factor within-subjects ANOVA for each task modality. (All results for all domains are on display in Fig. 2, in which the implicit level data are plotted as well for the sake of direct comparison across task modalities and levels.) Significant group differences were established through Bonferroni post-hoc tests with α at .01. For all ANOVAs, the precondition of sphericity was violated, such that multivariate test statistics were used. Furthermore, all variable distributions deviated significantly from normal distribution (Kolmogorow-Smirnow tests for normal distribution, all *p* < .05). However, simulation studies have demonstrated that one-factor ANOVAs are particularly robust against violations of the precondition normality (Blanca et al. 2017), given a large-enough sample size, roughly >30 or 40 (Ghasemi and Zahediasl 2012) – a condition easily met in our study. We therefore decided to proceed with parametric testing in spite of violations of normal distribution, yet adapted the significance level to α = .01 across analyses as a countermeasure to the increased risk of type I errors.

For the *Metaphor* task modality, multivariate tests established a significant effect for domain, *Wilks’ Lambda* = .317, *F*(3, 210) = 150.89, *p* < .01. The lowest average objectivity rates emerged for the domains of *Personal Preference* (8%) and *Social Convention* (9%), with no significant difference between the two. Post-hoc tests established that the average objectivity rate for *Morality* was significantly higher (35%) than that of both subjective domains. The domain of *Science* received the highest average objectivity rate (68%), differing significantly from all other domains.

For the *Disagreement* task modality, multivariate tests established a significant effect for domain, *Wilks’ Lambda* = .167, *F*(3, 210) = 349.22, *p* < .01. The lowest average objectivity rates emerged for the domain of *Personal Preference* (5%), which was significantly lower than the rate for *Social Convention* (18%). The average objectivity rate of *Morality* was significantly higher (45%) than that of both subjective domains (a one-sample *t*-test established, however, that this objectivity rate of *Morality* was still significantly below 50%, *t*(212) = −2.51, *p* < .05.) The domain of *Science* (79%) produced a significantly higher average level of objectivist intuitions.

For the *Theory 1* task modality, multivariate tests established a significant effect for domain, *Wilks’ Lambda* = .117, *F*(3, 210) = 526.12, *p* < .01. The lowest average objectivity rates emerged for the domains of *Personal Preference* (4%) and *Social Convention* (5%), with no significant difference between those two. Post-hoc tests established that the average objectivity rate for *Morality* was significantly higher (33%) than that of both subjective domains. The domain of *Science* elicited the highest average objectivity rate (88%), differing significantly from all other domains.

For the *Theory 2* task modality, multivariate tests established a significant effect for domain, *Wilks’ Lambda* = .280, *F*(3, 210) = 179.94, *p* < .01. The lowest average objectivity rate emerged for *Personal Preference* (28%). *Social Convention* (32%) elicited a significantly higher ratio of objectivist responses. Post-hoc tests established that the average objectivity rate for *Morality* was significantly higher (43%) than that of both ‘subjective’ domains. The domain of *Science* received the highest objectivity rate (84%), differing significantly from all other domains.

The results across tasks can be summarized as follows: The domain of *Science* consistently elicited predominantly objectivist intuitions; the domains of *Social Convention* and *Personal Preference* consistently elicited non-objectivist intuitions; the domain of *Morality* elicited predominantly non-objectivist responses. This last finding is in line both with our hypothesis and with previous research (e.g., Pölzler and Wright 2020; Sarkissian et al. 2011); however, this tendency of eliciting non-objectivist intuitions was significantly less pronounced than it was for the paradigmatically subjective domains of *Social Convention* and *Personal Preference*. Indeed, averaging across all explicit tasks, a significantly higher proportion of objectivist intuitions was found for *Morality* (*M* = 39%) than for *Social Convention* (*M* = 16%) and *Personal Preference* (*M* = 11%), respectively. This data pattern seems in accord with the hypothesis of non-objectivism-leaning pluralism, according to which people hold objectivist views regarding *some* moral issues, while holding a majority of non-objectivist views with respect to the totality of issues within the domain (see introduction). In what follows, however, we will describe two further findings that are exceptions to this general pattern: When ‘extreme’ moral items were analyzed as well as when participants’ classification of scenarios was used to score the disagreement task, an *objectivist* ratio of metaethical intuitions emerged.

#### (B2) ‘Extreme’ Moral Scenarios

In order to obtain an indicator for intuitions about ‘extreme’ moral scenarios, we analyzed the extreme item *Rape* (‘Is raping a woman wrong?’) separately in each of the three tasks *Metaphor*, *Disagreement* and *Theory 1* (*Theory 2* was not based on presenting scenarios, so no ‘extreme’ item could be analyzed separately). Because the extreme items were also part of the summary score of the domain of *Morality*, we avoided direct statistical comparisons between the general score and the ‘extreme’ items, as we did with the ANOVAs reported above. In order to still locate ‘extreme’ moral scenarios on the objectivist/non-objectivist spectrum relative to the other domains (Fig. 2, indicators “M(E)”), we tested their objectivist/non-objectivist levels individually via one-sample *t*-tests against the cut-off value of 50%, reporting confidence intervals.

The ‘extreme’ moral scenario in the *Metaphor* task yielded a ratio of 53% objectivist responses, which did not statistically differ from 50%, with 95% CI [46%, 60%], *t*(212) = .89, *p* = ns. The ‘extreme’ moral scenario in the *Disagreement* task yielded a ratio of 80% objectivist responses, which was significantly higher than 50%, with 95% CI [74%, 85%], *t*(212) = 10.81, *p* < .01. The ‘extreme’ moral scenario in the *Theory 1* task yielded a ratio of 68% objectivist responses, which was significantly higher than 50%, with 95% CI [62%, 74%], *t*(212) = 5.65, *p* < .01 (the average scores per task modality reported here are on display in Fig. 2, indicators “M(E)”). If the objectivist/non-objectivist ratio of the ‘extreme’ items of all three test modalities was aggregated, an average ratio of 67% resulted, which was significantly higher than 50%, with 95% CI [63%, 71%], *t*(212) = 7.98, *p* < .01.

On the basis of these findings, we conclude that ‘extreme’ moral scenario generally elicited overall objectivist intuitions relative to the cut-off value of 50%, which provides a contrast to the domain of morality as such, which elicited overall non-objectivist intuitions relative to the cut-off criterion of 50%.

#### (B3) Differences in Metaethical Intuitions Given Self-Classification of Scenarios

**Fig. 3:**
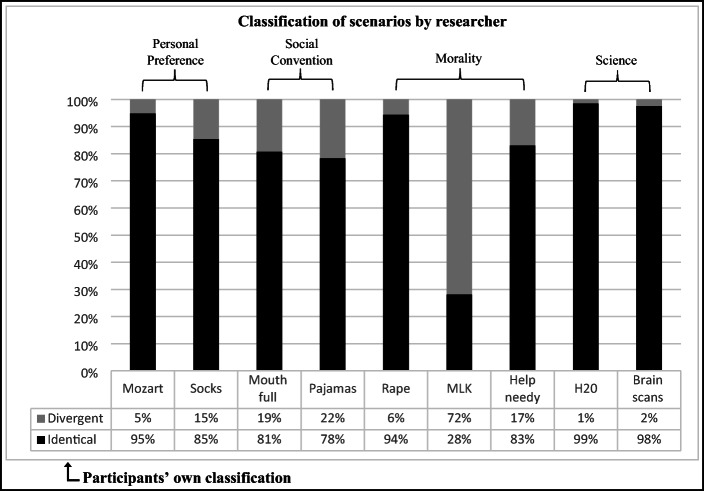
Ratio of identical vs. divergent domain classifications for each scenario

**Fig. 4:**
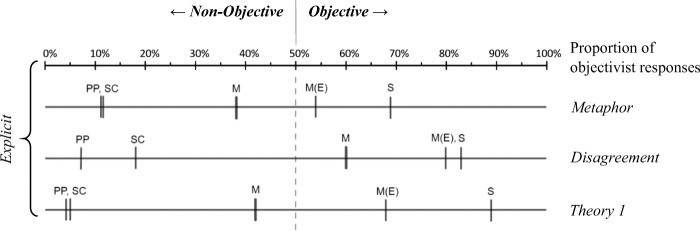
Personal Preference – Social Convention – Morality – Science: Objectivist/non-objectivist intuitions in three domains on the explicit level, re-scored based on self-classification. *Note:* Like Fig. 2, this figure places the domains on a spectrum of objectivist/non-objectivist intuitions for each task; however, in obtaining the present scores, participants own classification of issues was used in scoring intuition indices for each domain. For instance, in order to obtain a participant’s meta-intuition in the domain of *Morality*, precisely those (and only those) issues were scored which this particular participant had themselves classified as ‘moral’. This procedure could only be applied to those tasks which worked with particular scenarios. This was not the case for the implicit IAT tasks and the *Theory 2* task, and hence could only be applied to the three remaining explicit tasks: *Metaphor*, *Disagreement* and *Theory 1*. Post hoc tests established that all differences between domains are significant at *p* < .05, unless indicated by domain letters which are grouped together and merely separated by a comma. ‘Extreme’ moral items (indicator “M(E)”) have not been included in domain comparisons but have been tested individually against the cut-off value of 50%.

We had formulated the non-directional hypothesis that the ratio of objectivist/non-objectivist intuitions in the domain of *Morality* might change if only those items are scored which the participants themselves identified as pertaining to the domain of *Morality*. (Fig. 3 shows the ratio of whether the participant’s classification of each scenario was identical or divergent from our assignment of scenarios to domains; Fig. 4 graphically displays the scores recalculated with participants’ self-classification of scenarios). Indeed, a noteworthy shift emerged with respect to *Morality*: In the disagreement task, the sample now exhibited a 60% objectivist/non-objectivist ratio, which a one-sample *t*-test established to be significantly different from 50%, *t*(206) = 3.96, *p* < .01. In other words, the disagreement task elicited predominantly *objectivist* metaethical intuitions when participants’ own classification of scenarios was used. Three reasons might explain this swing towards objectivism in the disagreement modality: First, we know from past studies that disagreement tends to elicit stronger objectivist responses compared to other explicit task modalities (Pölzler and Wright 2020). Second, people might in fact have more objectivist intuitions on issues they themselves consider properly moral. Third, extreme contents such as ‘rape’ might be both more readily classified as moral *and* yield a higher rate of objectivist responses.

It was furthermore of interest that on average only 69% percent of moral scenarios were identified as such by participants. A significant proportion of moral scenarios was classified into the two ‘subjective’ domains: 15% of moral scenarios were perceived as pertaining to the domain of social convention, 16% as pertaining to personal preference; 0% of moral scenarios were classified as pertaining to the domain of science.^16^

It is worth emphasizing that the disparity between researchers’ classification and what the ‘folk’ considers to be moral issues differ enough to caution against making untested assumptions in that regard. While a number of researchers have been tempted to respond to data of classification disparities by attributing it to the indistinctness of folk understanding, research on qualitative responses suggests that people have a relatively solid intuitive grasp of the distinctions between these domains (Wright et al. 2013). This recommends the inclusion of a modality of analysis that uses the self-classification of scenarios by participants, as in the present study.

### Relation between Implicit and Explicit Levels (C)

#### (C1) Associative Relationships between Implicit and Explicit Tasks

Table 3 shows correlative relations among explicit tasks, implicit tasks, as well as between explicit and implicit tasks. Regarding the explicit level, the substantial positive correlations among the explicit tasks were to be expected based on previous research; the fact that correlations extend to the newly developed *Theory 2* task supports our prima facie case for the validity of this explicit test modality. Regarding the association of the two implicit tasks, the small but significant correlation of .24 is concordant with the assumption that they both measure the same construct (i.e. implicit metaethical intuitions).

**Table 3:** Correlations between implicit and explicit measures of metaethical intuitions across participants (*N* = 213)

Metaethical intuitions			1	2	3	4	5	6	7
Implicit	1	SC-M-S: *d*-score	–						
	2	PP-M-S: *d*-score	.24*	–					
	3	SC-M-S: extreme	–	–	–				
	4	PP-M-S: extreme	–	–	.19*	–			
Explicit	5	Metaphor	.07	.06	.03	.06.	–		
		– extreme	.00	−.05	.03	−.01.	–	–	
	6	Disagreement	.01	.03	.00	−.03	.30*	–	–
		– extreme	.08	.00	.05	−.03	–	–	–
	7	Theory 1	.02	.05	−.02	−.06	.37*	.52*	–
		– extreme	.04	−.06	.01	−.10	–	–	–
	8	Theory 2	−.01	.03	−.02	−.09	.25*	.28*	.30*

* *p* < .05

*Note*: Since ‘extreme’ *d*-score items were part of the item pool used to calculate the general *d*-scores, correlations between general and ‘extreme’ *d*-scores are not displayed. Similarly, the main explicit variables reported are summative scores which include the ‘extreme’ scenario, wherefore the correlations between them and the ‘extreme’ explicit variables are not displayed.

Regarding the relationships between explicit and implicit tasks, no substantive relations across the implicit/explicit distinction could be observed. In fact, not a single significant correlation emerged between the two implicit *d*-scores and the explicit tasks; this includes the *Theory 2* task, which consisted of an evaluation of the very same moral items as used in the IAT. To further investigate this surprising finding, we conducted 2 × 2 χ^2^-analyses by splitting objectivist from non-objectivist respondents differentially for explicit and implicit tasks. However, χ^2^ results also did not expose any deviations from expected cell-frequencies which would have suggested an associative relationship between explicit and implicit metaethical intuitions. Thus, contrary to hypothesis C1, both correlative and frequency analyses converged on finding no associative pattern or relationship between implicit or explicit metaethical (non-)objectivism.

### Consistency of Metaethical Intuitions and their Inter-Scenario Stability (D)

#### (D1) Consistency of Meta-Intuitions Regarding the Domain of Morality

Across the three established explicit tasks *Metaphor*, *Disagreement* and *Theory 1* only 5.2% of participants gave at least 80% objectivist responses to the moral scenarios presented, while 17.8% of participants responded with at least 80% non-objectivist responses to all of the moral scenarios.

With respect to the implicit tasks, consistency is arguably already part of the *d*-score. Given the general logic of our set-up, if a *d*-score in our ‘Metaethical Intuitions IAT’ differs relevantly from zero, this should be an indication that there is a systematic tendency towards objectivist or non-objectivist answers across a large number of items. To obtain a sense of the consistency of responses on the implicit level we therefore looked at what percentage of participants obtained a *d*-score that differed more than one standard deviation from zero, resulting in the following pattern: With respect to the PP-M-S IAT, only 0.9% of participants had a *d*-score that indicated consistent objectivist responses, while 65.3% exhibited consistently non-objectivist responses. Regarding the SC-M-S IAT, only 1.9% showed consistent objectivist responses, while 54.5% were consistently non-objectivist.

We might thus conclude that across explicit and implicit tasks, there is only a small minority of people who deliver consistent moral objectivist responses across tasks; and we might also say that this pattern seems to be more pronounced on the implicit level than it is on the explicit level (although caution is due, since the tasks and consistency criteria used were quite different). Furthermore, while there is also only a minority of people that consistently exhibit non-objectivist responses on the explicit level, a weak majority consistently tended towards non-objectivism on the implicit level.

#### (D2) Inter-Scenario Stability of Metaethical Intuitions

As has been mentioned in the sub-section describing the results for the ‘extreme’ moral items, there was significant variation across moral scenarios. As *t*-tests established, the scenario *Rape* (*M* = 70,0%, *SD* = 31.1%) elicited a significantly higher ratio of objectivist intuitions than *Martin Luther King* (*M* = 22.1%, *SD* = 32.0%), *t*(212) = 16.64, *p* < .01, as well as *Help the Needy* (*M* = 22.4%, *SD* = 31.1%), *t*(212) = 17.78, *p* < .01. In contrast, the objectivity ratios elicited by *Martin Luther King* and *Help the Needy*, respectively, did not differ significantly, *t*(212) = −.14, *p* = ns. Thus, the inter-scenario variation among the moral items could be attributed to the fact that the extreme moral item *Rape* differed significantly from the scenarios *Martin Luther King* and *Help the Needy*. Indeed, only the ‘extreme’ moral scenario exhibited the unique pattern of eliciting predominantly objectivist metaethical intuitions. The metaethical ‘pluralism’ of elicited intuitions across moral scenarios observed in our study could thus be reducible to a difference between moderate and extreme items.

## Discussion

We will first discuss the results of our efforts towards validating a new assessment of implicit metaethical intuitions, the ‘Metaethical Intuitions IAT’. Then we will elaborate on the main result of the implicit tasks. We offer an interpretation of our findings as evidence for moderate non-objectivism of implicit folk metaethical intuitions, which we take to be in line with the non-objectivist-leaning pluralism that has been claimed to characterize the folk’s explicit metaethical intuitions.

### Towards a Validation of a Novel IAT Procedure for Metaethical Intuitions

Our study proposed and piloted a novel IAT procedure for implicit metaethical intuitions, taking first steps towards developing valid tests of this form of cognition. IAT measures are an intuitive procedure to measure implicit cognition with considerable face validity, if domains and items transparently represent the constructs that researchers take them to represent. We take our domains and item sets to be transparent and representative in this sense. Indeed, the Single Category IAT procedure we proposed has an even simpler and more straightforward structure than standard IATs, given that only one target domain – *Morality* – is tested for whether it is associated rather with a paradigmatically objective or subjective domain. The domains on which we relied in construing our IAT dimensions and item sets have been well-defined and studied in existing research employing explicit tests of metaethical intuitions. On these grounds, we assume a solid prima facie case in favor of the validity of our procedure. We will, however, discuss in the following some possible alternative interpretations of the domain associations that manifested in the IAT tasks with our sample.

In terms of construct validity, a valid measurement of implicit metaethical intuitions would be expected to be related, in a moderate degree, with other measures of metaethical intuitions (Nosek et al. 2005; Strauss and Smith 2009; Campbell and Fiske 1959), such as well-established tasks for explicit metaethical intuitions. Indeed, we observed two parallels in the result patterns on the implicit and explicit level (cf. hypothesis C2) which deserve mention as evidence of such convergent validity:

1. Both on the implicit and on the explicit level, people predominantly showed non-objectivist intuitions with respect to the domain of *Morality*.
2. On the explicit level, the domain of *Morality* elicited a higher proportion of objectivist responses compared to the domains of *Personal Preference* / *Social Convention*, yet a lower proportion of objectivist responses than towards the domain of *Science*. Furthermore, as evidence from analyses based on participants’ self-classification of scenarios showed, the domain of *Morality* had more overlaps with the domains of *Personal Preference* / *Social Convention* than with *Science*. Concerning the implicit level, this pattern was echoed in the Theory 2 task, in which participants evaluated the item sets that were administered on the implicit level. Regarding the IAT results themselves, the significant but moderate effect sizes of IAT *d*-scores suggest a similar finding of the domain of *Morality* being perceived as non-objective, yet to a moderate degree (differing therein both from the objective nature of the domain of *Science* and the strongly subjective nature of the domains of *Personal Preferences* and *Social Conventions*, all of which may be plausibly presumed).

These two points, indicating a parallelism of result patterns on the implicit and explicit level, speak in favor of hypothesis C2 and the construct validity of the ‘Metaethical Intuitions IAT’ as a measure of metaethical cognition and attitudes. However, we also had hypothesized (C1) that implicit and explicit tasks would be directly associated. Contrary to expectation, no correlative relations between implicit and explicit task results emerged. In this respect, we therefore found no evidence of convergent validity. This lack of significant correlations also held for the local *d*-score of ‘extreme’ moral items. Frequency analyses could also not detect any associative relations between explicit and implicit tasks, nor did further exploration of the data unearth any non-linear relationships.

Moreover, a divergent pattern of results on the explicit and implicit level emerged with regard to extreme moral issues. The extreme moral scenario (contained in the task modalities *Metaphor*, *Disagreement*, *Theory 1*) elicited overall objectivist intuitions, while moderate scenarios elicited overall non-objectivist intuitions. Contrary to our hypotheses (A2, C2), this pattern was not echoed on the implicit level, as *d*-scores regarding ‘extreme’ moral items remained fully consistent with the non-objective pattern of the general IAT *d*-scores.

Given the considerable face validity of the ‘Metaethical Intuitions IAT’ and the two abovementioned parallels in explicit and implicit result patterns, the lack of correlations and the divergence on ‘extreme’ moral issues might also be taken to signal that implicit cognition of moral concepts is relatively independent from cognition on explicit levels. Caution is due with this line of thought, however, as this conclusion presupposes the validity of the implicit tasks and assumes that there are no alternative explanations for the domain associations we observed in our sample.

Given the tentative evidence and the early state of research on this topic, it seems appropriate to adopt a minimal interpretation of what the ‘Metaethical Intuitions IAT’ tasks measured. Instead of interpreting *d*-scores as full-fledged implicit metaethical intuitions, we suggest it is advisable at this point to take them as indicators for how domains of terms are perceived as more or less semantically related. Yet, we would like to note that even this more moderate interpretation exhibits significant explanatory power regarding implicit metaethical thought regarding objectivism and subjectivism. It suggests that, by default, the folk more strongly associate moral terms with terms describing *Personal Preferences* or *Social Conventions*, i.e. domains philosophers (as well as the folk) typically identify as ‘subjective’ domains. We will therefore retain talk of objectivism/subjectivism regarding IAT results in the rest of this paper, but do so in cognizance of the qualification just discussed.

Beyond the recommended minimalist interpretation, we still consider it plausible that the observed domain associations are substantially driven by the perceived objectivity/subjectivity of domains rather than other factors. One problem threatening this stronger interpretation, however, is that concepts and terms can be associated based on various features, with objectivity/subjectivity being only one among many possible dimensions that could be at play. As Fiedler et al. (2006) formulate this problem: There might be different types of associations between IAT dimensions, but they all result in one *d*-score that quantifies the implicit attitude. An alternative explanation for the association of domains that emerged in our study might be, for example, ‘relatedness to personal or human affairs’. On that logic, the close association of *Moralit*y with terms of *Personal Preference* and *Social Convention* can be explained by the fact that these domains are all related to human social or personal affairs, whereas scientific terms on the contrary are more associated with clean and sterile investigations of the objective/material world. Similarly, it might be hypothesized that *Morality* / *Personal Preference* / *Social Convention* are all ‘socially normative domains’, i.e. domains of lived choices or experiences that directly impact our lives. Although science is something people (scientists) do that can have implications for our lives, it is less directly related to our everyday choices, decisions, values and norms. On yet another variation of this line of thought, ‘methodological terminology’ might constitute a further alternative to our preferred explanation of what underpins the observed domain associations. Indeed, *Morality* / *Personal Preference* / *Social Convention* do not include any methodological terminology, whereas the terms we used to represent the domain of *Science* were in part straightforwardly methodological (e.g., ‘experimentation’ or ‘searching for correlations’).

Yet, the design of our study makes some of these alternative explanations less likely than our proposed interpretation of the association as expressive of subjectivism/objectivism. For instance, the labels “Subjective” / “Objective” were placed next to the respective domain labels in the IAT set-up in order to make the dimension of interest salient to participants. Nonetheless, we suggest that future studies into implicit metaethical cognition should seek to decrease the likelihood of these alternative explanations further, for instance by highlighting the dimension of interest even more effectively, or by avoiding – as much as this is possible – methodological or technical jargon in operationalizing the item set for the domain of science.

However, not all such confounds can be, or even should attempted to be, eliminated or cleanly separated from the dimension of objectivity/subjectivity. For instance, the fact that – in contrast to science – the domains of *Morality*, *Social Conventions* and *Personal Preferences* are concerned with human affairs rather than the objective/material world is an integral feature of these domains. Researchers must take into account that such domain features and differences might to some extent be an *explanation* for metaethical (non-)objectivism rather than a confound, in that morality’s concern with human affairs might be a reason *why* the domain is tendentially perceived as non-objective in contradistinction to science. Such inseparability does in no way exclude the possibility or benefit of methodically studying such interconnected variables in their relation to the dimension of objectivity/subjectivity. Indeed, we believe that a systematic variation of the set-up and item sets of the IAT procedure we have introduced provides a methodological basis for this type of research, thereby opening up various avenues for further research.

### Main Results of the ‘Metaethical Intuitions IAT’ Procedure

With regard to philosophers’ burden of proof argument for moral objectivism, our study’s main result is that akin to findings on the explicit level, non-objectivist intuitions about *Morality* are dominant on the implicit level as well.^17^ Two caveats to this general statement should be noted, however. First, regarding the explicit level, the domain of *Morality* did in fact trigger ~40% of objectivist responses. Hence, although there is a clear tendency of the folk towards non-objectivist attitudes on the explicit level, there also is a substantial proportion of objectivist intuitions that is significantly higher than that yielded by the domains *Personal Preference* and *Social Convention*. However, all of this is well captured by the interpretation that the folk exhibits non-objectivism-leaning metaethical pluralism in their explicit metaethical intuitions, a hypothesis suggested as characterizing the findings of recent research on explicit metaethical intuitions generally (Pölzler and Wright 2020). Second, regarding the implicit level, the *d*-scores that emerged were of moderate effect size. We thus interpret our findings summarily as evidencing that non-objectivism-leaning pluralism most adequately reflects the composition of lay people’s explicit metaethical intuitions, while implicit metaethical cognition suggests consistent yet moderate non-objectivism.

*Explicit pluralism, implicit non-objectivism: The involvement of explicit cognition in the perceived objectivity of ‘extreme’ moral issues.* In line with past research, we observed that the metaethical pluralism among moral scenarios of explicit tasks seems to be largely driven by the difference between moderate and ‘extreme’ moral scenarios. The extreme moral scenario ‘Is raping a women wrong?’ consistently triggered a majority of objectivist intuitions among all explicit tasks that employed this scenario, contrary to the non-objectivist response elicited by the other, moderate items. We take this to suggest that the pluralist variation in explicit metaethical intuitions was largely driven by the feature of moderacy vs. extremity of the contents of scenarios. As we noted before, this pattern did not manifest in the responses to extreme items in the newly developed IATs, insofar local *d*-scores calculated with the extreme items ‘rape’ and ‘genocide’ remained firmly consistent with the general non-objectivism on the implicit level. We would now like to further elaborate on this point, as it illustrates important respects in which the nature of explicit and implicit metaethical cognition might fundamentally differ.

First, what is understood by ‘extreme’ moral items? For the purposes of this study, we relied on a rather sketchy and pre-theoretical definition of ‘extremity’, roughly referring to intentionally inflicted extreme harms (Schein and Gray 2015), viz. ‘rape’ and ‘genocide’. More precisely, ‘extremity’, could be defined as involving actions that directly, violently and seriously impinge on a victim’s integrity with a strong bodily component – somewhat similar to Greene et al.’s (2001) definition of “personal harm”. A higher level of extremity in this sense can explain why a scenario like rape would trigger an overall objectivist response in explicit tasks. Our finding that ‘extreme’ items elicit non-objectivist responses on the implicit level might then suggest that grasping the objectivity of the wrongness of rape requires explicit moral deliberation and reflection above and beyond, and perhaps even contrary to the more immediately available processing outputs of implicit metaethical cognition.

However, there are alternatives to this extreme-content explanation, as other item-features such as non-ambiguity and context-independence might also elicit higher rates of objectivist intuitions. While it is unlikely that people associate concepts like rape or genocide with situations in which these actions would be morally justified, there are easily imaginable circumstances in which bravery, honesty, or generosity may or may not be required. If the latter set of moral concepts were disambiguated by specifying a situation in which it is clear that acts or character traits of their type are morally required, it seems possible that also some less extreme items might show more objectivist response patterns. Furthermore, issues like ‘rape’ and ‘genocide’ might also be argued to display the feature of being absolutely/universally wrong; it is conceivable that actions that do not entail extreme bodily harm but are universally and unambiguously wrong, such as ‘cheating on your partner with their best friend’, also evoke more objectivist intuitions. Further research may more specifically examine our tentative findings regarding the centrality of extremity by directly testing it against such alternative explanations. In our view, this would be part of a broader effort to better understand the factors driving the pluralism of explicit meta-intuitions regarding issues in the moral domain. If this is better understood, we would also better understand which cognitive processes bring about the notable contrast in response to ‘extreme’ moral items that we observed, namely an objectivist response in explicit tasks and a non-objectivist response on the implicit level.

That said, it is possible that the relation between the level of ‘extremity’ and objectivist intuitions is largely or fully mediated by factors that have been found to promote such intuitions in previous studies. This seems particularly plausible for two such factors: strength of agreement (people likely agree to a high extent that actions such as rape and genocide are wrong), and perceived consensus (there is high societal consensus that these actions are wrong) (e.g., Ayars and Nichols 2019; Beebe 2014; Beebe and Sackris 2016; Goodwin and Darley 2008, 2012; Heiphetz and Young 2017; Pölzler and Wright 2020; Wright and Pölzler forthcoming). If these are the case, lay people’s objectivist intuitions about ‘extreme’ scenarios would not be generated by the objective moral content of these items but rather by contingent individual and societal facts about them. We therefore want to emphasize that, given the possibility of alternative explanations, our notion of extremity is only a tentative explanation of why the ‘extreme’ moral scenario produced more objectivist responses in explicit tasks. Importantly, also these deflationary explanations of ‘extremity’-related objectivism in explicit tasks are aspects in which explicit and implicit metaethical cognition might differ. The processing of strength of agreement and relative consensus might require explicit cognition that is bypassed in IAT tasks – a further reason to think that the IAT procedure here presented might be a fruitful supplementary tool in studying the full spectrum of metaethical intuitions.

#### Limitations

Given the nature of IAT methodology, it is not possible to include the evaluation of entire propositions or sentences among the items (unlike scenarios of explicit tests of metaethical intuitions, where this is possible). What can be tested is only the association between sets or domains of terms or concepts. However, there are reasons to think that this is not a serious limitation. First, most metaethicists assume that the metaphysical commitments of moral concepts remain stable across varying objects of application, e.g., “wrong” refers to an objective property in “rape is wrong”, “murder is wrong”, “cheating in bord games is wrong”, etc. (e.g., Brink 1989; Huemer 2005; for discussion see Gill 2009). In current metaethical debates, philosophers typically consider the status of *all* moral language and thought, thus including not only propositions but also concepts. Second, one also often finds philosophers explicitly talking about concepts rather than propositions; for instance, Brink defines metaethics as “conceptual or logical analysis of fundamental moral concepts.” (Brink 1989: 3) For these reasons, we think that there are fruitful ways in which the IAT methodology can be employed to assess metaethical intuitions.

Furthermore, the results of our study may not be widely generalizable (just as the results of most previous studies on explicit metaethical intuitions, see Pölzler 2018). Even though we strived for representative heterogeneity within our pool of moral items, our results may be crucially contingent on the selection of particular items that we used (for a discussion of this problem, see Clark 1973; Judd et al. 2012). Finally, as all of our participants were US citizens, we also cannot exclude that people from non-Western cultures may differ in their implicit metaethical intuitions (see Henrich et al. 2010).

### Implications for the Philosophical Debate

According to philosophers’ widely accepted burden of proof argument for moral objectivism (e.g., Brink 1989; Dancy 1986; Huemer 2005), lay people intuitively affirm that morality is objective. Criticisms of this empirical assumption have often been dismissed on the grounds that this claim refers to implicit rather than explicit metaethical intuitions (e.g., Björnsson 2012; Brink 1989; Enoch 2005, 2014). The goal of this study was to contribute to assessing this reply directly by furthering a more comprehensive empirical understanding of actual folk metaethical attitudes on the implicit level.

The ‘Metaethical Intuitions IAT’ employed in this study yielded preliminary evidence against the hypothesis that lay people are implicit moral objectivists. Even on an implicit level, intuitions seem to predominantly tend to non-objectivism. That is, lay people implicitly process the majority of moral concepts as akin to conventions or preferences. This finding (which is roughly in line with Pölzler et al. under review, Theriault et al. 2017 as well as much recent research on the explicit level) is of considerable philosophical importance.

Metaethicists who have insisted that only or mainly implicit intuitions matter have presumably done so because they distrust people’s ability to access or articulate these intuitions. In the introduction, for example, we found Enoch dismissing the explicit beliefs and statements of “sophomores” (Enoch 2005: 773, footnote 31; as well as Björnsson 2012); and, in a later publication, he directly states that people’s explicit intuitions are largely mistaken: “You may think that you’re a moral relativist or subjectivist – many people today seem to. But I don’t think you are” (Enoch 2014: 193). Contrary to this supposition, our results suggest that people’s explicit intuitions might track their deeper metaethical commitments fairly well (albeit not perfectly).

Even more importantly, most metaethicists may also have misjudged the content of people’s intuitions. Our results give us additional reason to be skeptical of metaethicists’ dominant assumption that the folk are drawn towards moral objectivism, and consequently, of any philosophical arguments that rely on this assumption, such as, first and foremost, the burden of proof argument (for discussion see, e.g., Davis 2021; Hopster 2019; Pölzler 2018).

As has been explained in the introduction, the assumption that non-objectivists bear the burden of proof — that unless they have presented strong arguments in their favor we should stick with our purported objectivist starting point — has significantly shaped metaethical debates. It has led to a situation in which non-objectivists dominantly try to come up with arguments in their favor, and objectivists dominantly try to refute these arguments. But if the burden of proof argument’s empirical assumption of implicit folk moral objectivism turned out to be implausible, then this dialectic would have to be rethought. Objectivists could no longer be taken to win the argument by default. In addition to increased pressure to engage with the arguments of non-objectivists, philosophers advocating for objectivism would be required to present a stronger positive case for the existence of objective moral truths. To the extent that the objectivist position rests on a presumption of general folk metaethical objectivism, this would make it significantly more justifiable to adopt non-objectivist theories, i.e., theories that deny such truths.

Our results may contribute to such a reevaluation of the traditional dialectic of the moral objectivism debate. As we have indicated, we take a moderate non-objectivism on the implicit level and a non-objectivism-leaning pluralism on the explicit level to be most characteristic of the folk’s metaethical commitments. In any case, we hope that our novel procedure for measuring implicit intuitions about moral objectivity — the ‘Metaethical Intuitions IAT’ — serves as a catalyst for more extensive research into implicit metaethical cognition.

## APPENDIX – IAT for metaethical intuitions: Categories and items

### “Morality”

doing good deeds

injustice

inflicting pain

helping the needy

ethical values

vicious

being honest

bravery

expressing empathy

rape

genocide

acting generously

### “Science (Objective)”

conducting measurements

reliable methods

exact calculations

facts

objective truth

reporting correctly

hard evidence

well-tested thesis

reproducible results

experimentation

searching for correlations

being clear and analytic

### “Social Convention (Subjective)”

being polite

table manners

professional dress

greeting

fitting in

social expectations

being on time

drinking age

speed limit

national holiday

required schooling

local slang

### “Personal Preference (Subjective)”

favorite food

taste in music

hobbies

slight aversion

clothing style

political opinion

attractive looks

best movies

choosing a college major

exercising routine

favorite TV show

being a hippie

## Footnotes

1 Most metaethicists who have tried to defend the burden of proof argument’s empirical assumption by stressing the importance of implicit intuitions have not done so in direct reply to empirical research. They rather worried about anecdotal evidence in favor of folk moral non-objectivism, in particular, as Enoch’s quote shows, among students. It is highly likely, however, that these metaethicists would raise the very same criticism against empirical studies that seem to cast doubt on their assumption. A number of researchers have interpreted the metaethicists in this way and have hence related them to these empirical studies (e.g., Pölzler et al. under review; Zijlstra forthcoming). In fact, at least some proponents of the burden of proof argument have themselves framed their implicit intuitions reply as addressing, among other things, psychological research on folk metaethics (e.g., Björnsson 2012).

2 For example, the study by Zijlstra only involved a small number of moral items (which makes it difficult to detect pluralism); and the study by Pölzler, Zijlstra and Dijkstra made use of metaethical vocabulary that might prompt explicit thinking.

3 The moral objectivists’ burden of proof argument makes an empirical assumption about the metaethical *attitudes* of the folk. Thus, we confine our present aim to complementing research investigating metaethical attitudes and bracket, for the time being, any questions if and how these attitudes are related to behaviors. Furthermore, unlike IATs that aim at assessing prejudices, social desirability seems a marginal issue in our task for metaethical intuitions, if only because it is highly unlikely that participants grasp what this IAT might actually be assessing. The dangers of faking and strategic processing (Fiedler et al. 2006) seem therefore negligible as well. Finally, we will critically discuss and address the most pivotal issues with IATs in this paper, such as response consistency, validity, and the difficulty of making inferences about attitudes based on the observation of mere associations.

4 We are aware that philosophers have defended the objectivism of morality with reference to *mathematics* (e.g., Clarke-Doane 2020; Franklin 2004; Peacocke 2004), but it is worth pointing out that many currently influential moral objectivists like Boyd 1988; Brink 1989; Railton 1986; etc. explicitly model morality after *science*, including some of the staunchest defenders of the burden of proof argument (such as Brink). Moreover, in investigating folk attitudes, the domain of science seems a more apt objectivist contrast than that of mathematics, assuming that a non-technical understanding of facts is closer to scientific findings than mathematical conclusions. This stance is reflected in recent research, too: Investigating the ‘preference’ vs. ‘fact’ distinction as it relates to morality, Theriault et al. (2017) used experimental science as the contrasting objective domain, as did much other research on folk metaethics (e.g., Goodwin and Darley 2008; Wright et al. 2013).

5 For the sake of clarity, we will capitalize and italicize terms with which we refer to a domain in general, e.g., *Morality*. However, if the domain is operationalized as a label in the IAT, it will be capitalized and put in quotation marks, e.g. “Morality”.

6 It deserves mention that we also preregistered a further hypothesis regarding the relation between the implicit and the explicit level, namely that there might be an ordinality of results among the explicit tasks, depending on their ‘level of explicitness’, and correspondingly stronger associations with the implicit tasks (Pölzler et al. 2019). However, we now concede that there is no meaningful ‘ordinality of explicitness’ among the explicit tasks we used.

7 The time estimate of 30 min includes all implicit and explicit tasks, the automatic relocations between online platforms, as well as the download and installation of the IAT software “Inquisit Lab 5”.

8 If either (a) or (b) was the case for a participant, we did not reimburse this person on the basis of their insufficient effort response. Regarding (c), we withheld reimbursement only if accuracy was below 50% and the response pattern indicated that the person was skipping through portions of the task.

9 Our IAT set-up in Inquisit scripts – including all parameters, items, etc. – is available for download in files attached to our preregistration (Pölzler et al. 2019).

10 This increased variety of experimental stimuli has recently been pushed on theoretical grounds. As described in more detail in Pölzler (2018), most existing studies have so far only tested statements that involve “thin” moral concepts and action types (while a number of objectivists have actually also been interested in virtues, e.g., Marshall 2018). For studies which have broadened the scope of their stimuli see Goodwin and Darley 2012; Pölzler and Wright 2020; Wright and Pölzler forthcoming.

11 Typically, IAT tasks have two concepts for each, the target dimension and the attributive dimension. However, we were only interested where the domain of *Morality* falls on the spectrum of subjectivity/objectivity, and there is no straightforward opposing domain to the domain of *Morality*. We therefore decided to use a procedure known as ‘Single-Category IAT’ (Karpinski and Steinman 2006). Unlike the standard IAT task, the ‘Single-Category IAT’ only measures the association of one target concept – in our case “Morality” – with two concepts on the attributive dimension.

12 At the same time, the two domain labels of the attributive dimension appeared in the same configuration for each participant. However, whereas in the SC-M-S IAT, the label “Science (Objective)” remained on the right and the label “Social Convention (Subjective)” on the left, this was reversed in the PP-M-S IAT, in which the label “Science (Objective)” remained on the left and the label “Personal Preference (Subjective)” on the right.

13 *d*-scores were calculated by subtracting the mean reaction time in the compatible condition of the IAT (as defined through the directional hypothesis) from the mean reaction time of the incompatible condition and standardizing the result (division by standard deviation of all reaction times). Like Karpinski and Steinman (2006), we removed non-response trials from *d*-score calculations and replaced error responses with the mean latency of the participant plus a 400 ms penalty.

14 Given that two IATs were administered in sequence, we investigated potential practice effects which might arise from the fact that item sets of those domains common to both IATs (viz. *Science* and *Morality*) would recur in the IAT presented second place. Regarding the SC-M-S IAT, a significant sequence effect emerged for the order of presentation. If the SC-M-S IAT was presented first place, the observed *d*-score for this IAT was on average significantly higher (*d* = .63) than for participants which completed it second place (*d* = .41), *t*(203) = −4.63, *p* < .01. However, the *d*-scores of both first place and second place presentations of this IAT task remained significantly different from 0 and remained conformant with the generally observed non-objectivist trend (one-sample *t*-tests all *p* < .01). Regarding the PP-M-S IAT, no significant sequence effect emerged. Thus, insofar there were practice effects arising from repeated domain presentation due to the sequential administration of two IATs, these had no substantial impact on the reported overall pattern of results.

15 These extreme items were included in calculating the general *d*-scores, as we take it that some extreme examples are an integral part of the diverse domain *Morality*.

16 It is noteworthy in this regard that only 28% considered the question whether Martin Luther King was brave to be a properly moral issue. This result is in line with past research using items that employ virtue concepts (Pölzler and Wright 2020); however, studies employing explicit tasks have still relied on such items as they clearly involve a concept regarded as moral in nature from the perspective of most philosophers.

17 Our results are thus remarkably in line with the main finding of Theriault et al.’s (2017) study into the neurological basis of metaethical cognition, namely that moral issues are neuro-cognitively processed more like preferences rather than scientific facts.
